# Supplementary material for: MicroRNA Expression Prior to Biting in a Vector Mosquito Anticipates Physiological Processes Related to Energy Utilization, Reproduction and Immunity
Source: Insects. 2023 Aug 10;14(8):700. doi: 10.3390/insects14080700 (PMC10455316; doi:10.3390/insects14080700)

Supplementary Materials File for:
MicroRNA expression prior to biting in a vector mosquito anticipates physiological processes related to energy utilization, reproduction, and immunity.

Sarah Marzec, Alden Siperstein, Angela Zhou, Christina M. Holzapfel, William E. Bradshaw, Megan E. Meuti, Peter A. Armbruster*
*Author for correspondence: paa9@georgetown.edu

Table S1. List of the 144 unique mature miRNAs combined from Hong et al. (2014) and miRbase. miRNAs from Hong et al. are named additionally with position information (i.e. super contig location) and miRNAs from miRbase are labeled with identifiers from the miRbase database (“MIMAT”).

**Mature miRNA**	**Sequence**	
Cpp-bantam_supercont3.65:199694-199763	TGAGGTAGTTGGTTGTATAGT	
Cpp-miR-1-3p_supercont3.78:246202-246281	TGGAATGTAAAGAAGTATGGAG	
Cpp-miR-2-3p-1_supercont3.366:116579-116662	TATCACAGCCAGCTTTGAAGAG	
Cpp-miR-2-3p-2_supercont3.366:116853-116935	TATCACAGCCAGCTTTGAAGAGC	
Cpp-miR-7_supercont3.1:3357343-3357422	TGGAAGACTAGTGATTTTGTTGT	
Cpp-miR-8_supercont3.40:815854-815935	TAATACTGTCAGGTAAAGATGTC	
Cpp-miR-9_supercont3.1009:114890-114972	TCTTTGGTTATCTAGCTGTA	
Cpp-miR-10-3p_supercont3.12:95990-96072	CAAATTCGGTTCTAGAGAGGTTT	
Cpp-miR-11-3p_supercont3.153:639659-639743	CATCACAGTCTGAGTTCTTGCT	
Cpp-miR-12-3p_supercont3.153:639666-639741	TGAGTATTACATCAGGTACTGGT	
Cpp-miR-13_supercont3.366:116983-117072	TATCACAGCCATTTTGACGAGTT	
Cpp-miR-14-3p_supercont3.676:52241-52327	TCAGTCTTTTTCTCTCTCCTAT	
Cpp-miR-31_supercont3.559:256532-256609	TGGCAAGATGTTGGCATAGCTGA	
Cpp-miR-33_supercont3.1258:69328-69403	GTGCATTGTAGTTGCATTGCA	
Cpp-miR-71_supercont3.366:117493-117583	AGAAAGACATGGGTAGTGAGAT	
Cpp-miR-79-3p_supercont3.83:80579-80662	TAAAGCTAGATTACCAAAGCAT	
Cpp-miR-87_supercont3.431:379743-379819	GTGAGCAAATTTTCAGGTGTGT	
Cpp-miR-92a_supercont3.722:174902-174982	TATTGCACTTGTCCCGGCCTAT	
Cpp-miR-92b_supercont3.722:164904-164984	AATTGCACTTGTCCCGGCCTGC	
Cpp-miR-100_supercont3.4:271404-271484	AACCCGTAGATCCGAACTTGTG	
Cpp-miR-124_supercont3.8:2074719-2074803	TAAGGCACGCGGTGAATGCCAA	
Cpp-miR-125_supercont3.4:280965-281043	TCCCTGAGACCCTAACTTGTGA	
Cpp-miR-133-3p_supercont3.1189:55672-55785	TTGGTCCCCTTCAACCAGCTGT	
Cpp-miR-137-3p_supercont3.1714:27556-27636	TATTGCTTGAGAATACACGTAG	
Cpp-miR-184-3p_supercont3.567:240302-240382	TGGACGGAGAACTGATAAGGGC	
Cpp-miR-190-1_supercont3.181:347902-347986	AGATATGTTTGATATTCTTGGTTG	
Cpp-miR-210-3p_supercont3.549:157647-157724	CTTGTGCGTGTGACAACGGCTAT	

Cpp-miR-252-1_supercont3.1787:6793-6867	CTAAGTACTAGTGCCGCAGGAG
Cpp-miR-263_supercont3.219:351799-351880	AATGGCACTGGAAGAATTCACGG
Cpp-miR-275_supercont3.291:329765-329845	TCAGGTACCTGAAGTAGCGCG
Cpp-miR-276-3p-1_supercont3.136:340857-340942	TAGGAACTTCATACCGTGCTCT
Cpp-miR-277_supercont3.36:1153726-1153817	TAAATGCACTATCTGGTACGACA
Cpp-miR-278-3p_supercont3.16:1026152-1026243	TCGGTGGGACTTTCGTCCGTTT
Cpp-miR-279-3p_supercont3.19:1441114-1441197	TGACTAGATCCACACTCATTAA
Cpp-miR-281-1_supercont3.1661:13247-13325	AAGAGAGCTATCCGTCGACAGT
Cpp-miR-283_supercont3.57:559440-559549	CAATATCAGCTGGTAATTCTGGG
Cpp-miR-285-3p_supercont3.98:262280-262362	TAGCACCATTCGAAATCAGTAC
Cpp-miR-305_supercont3.291:339122-339204	ATTGTACTTCATCAGGTGCTC
Cpp-miR-306_supercont3.83:80431-80502	TCAGGTACTGAGTGACTCTCAG
Cpp-miR-308_supercont3.98:764123-764204	CGCGGTATATTCTTGTGGCTTG
Cpp-miR-309-3p_supercont3.145:66026-66116	TCACTGGGCATAGTTTGTCGC
Cpp-miR-315_supercont3.438:61916-61999	TTTTGATTGTTGCTCAGAAAGCC
Cpp-miR-316_supercont3.496:152458-152539	TGTCTTTTTCCGCTTACTGCCG
Cpp-miR-317-3p-1_supercont3.36:1133161-1133241	TGAACACAGCTGGTGGTATCT
Cpp-miR-375-1_supercont3.4:25124-25212	TTTGTTCGTTTGGCTCGAGTTA
Cpp-miR-932-3p_supercont3.261:301401-301481	TGCAAGCAATGTGGAAGTGA
Cpp-miR-957-3p_supercont3.787:29537-29622	TGAAACCGTCCAAAACTGAGGC
Cpp-miR-965-3p_supercont3.48:484122-484208	TAAGCGTATAGCTTTTCCCATT
Cpp-miR-970-3p_supercont3.495:35917-36003	TCATAAGACACACGCGGCTAT
Cpp-miR-980-3p_supercont3.263:352870-352954	TAGCTGCCTTGTGAAGGGCTTA
Cpp-miR-981_supercont3.431:151310-151408	TTCGTTGTCGACGAAACCTGCA
Cpp-miR-988-3p_supercont3.791:14282-14361	CCCTTGTTGCAAACCTCACGC
Cpp-miR-989-3p_supercont3.315:321306-321394	TGTGATGTGACGTAGTGGTAC
Cpp-miR-993_supercont3.12:55477-55565	TACCCTGTAGTTCCGGGCTTTT
Cpp-miR-996_supercont3.19:1437000-1437085	TGACTAGATTACATGCTCGT
Cpp-miR-998_supercont3.153:639515-639597	ACTGAATTCTCGTGGGTCTGCA
Cpp-miR-999-3p_supercont3.14:96865-96948	TGTTAACTGTAAGACTGTGTCT
Cpp-miR-1000_supercont3.153:102798-102891	ATATTGTCCTGTCACAGCAGT
Cpp-miR-1174_supercont3.86:865891-865964	CTGGGTATTTTAGATCATCGGC
Cpp-miR-117_supercont3.86:866106-866187	AAGTGGAGTAGTGGTCTCATCG
Cpp-miR-1889_supercont3.57:562539-562677	TAATCTCAAATTGTAACAGTGG
Cpp-miR-1890_supercont3.64:982778-982862	TGAAATCTTTGATTAGGTCT
Cpp-miR-1891_supercont3.829:180333-180418	TGAGGAGTTAATTTGCGTGTTT
Cpp-miR-2941-3p-1_supercont3.5:753632-753715	TAGTACGGCTAGAACTCCACGG
Cpp-miR-2951-1_supercont3.1464:4408-4477	GAAGAGCTCAGCACGCAGGGGTG
Cpp-miR-2952-3p_supercont3.5:753920-753983	TAGTACGGCCATGACTGAGGGC

Cpp-miR-iab-4_supercont3.12:681153-681228	ACGTATACTGAATGTATCCTGA
Cpp-miR-2779-1_supercont3.1527:60074-60167	ATATCCGGCTCGAAGGACCA
Cpp-miR-2796-3p_supercont3.1234:55494-55572	GTAGGCCGGCGGAAACTACTTGC
Cpp-miR-2840_supercont3.830:78060-78155	TAGGAACTGGAAGAAGAGGAGG
Cpp-miR-2942_supercont3.277:323072-323166	TATTCGAGACTTCACGAGTTAAT
Cpp-miR-2944b_supercont3.145:66345-66420	GAAGGAACTCCCGGTGTGATATA
Cpp-miR-2945_supercont3.4:184409-184491	TGACTAGAGGCAGACTCGTTTA
Cpp-miR-2981_supercont3.772:7842-7925	CCGGGCCGGGCGGGCGGG
Cpp-miR-3781_supercont3.1005:38090-38187	TAAGTGATTGATCGATCGTGGAT
Cpp-miR-4682_supercont3.1:1608156-1608242	TCTGAGTTCCTGGAGCCTGGTCT
Cpp-miR-4448_supercont3.461:292111-292200	GGCTCCTTGGTCTAGGGGTA
Cpp-miR-307_supercont3.16:157813-157891	TCACAACCTCCTTGAGTGAGCGA
Cpp-miR-493-3p_supercont3.147:509902-509990	TGAAGGTCCTACTGTGTGCCAGG
Cpp-miR-929-3p_supercont3.60:858168-858245	CTCCCTAACGGAGTCAGATTG
Cpp-miR-1290_supercont3.383:279453-279536	TGGATTTTTGGATCAGAGA
Cpp-novel-miR1_supercont3.64:982778-982859	AGAGCTAATTGGAGACTTCTTG
Cpp-novel-miR2-1_supercont3.360:171826-171903	GTGTCCTGTCACGGTCGCCA
Cpp-novel-miR3-3p_supercont3.112:3529-3623	GTTTGAACTTGATCCGCGGCTGA
Cpp-novel-miR4_supercont3.153:639486-639575	GTGCTTTTCGTTGGAACTTG
Cpp-novel-miR5_supercont3.316:156915-156993	AATTAGAAATCACACAAACGTT
Cpp-novel-miR6-3p_supercont3.1403:25947-26028	TAGGGAAACAGATTGGCCAATG
Cpp-novel-miR7-3p-1_supercont3.1002:30216-30294	ACATCGCGTGTCGTTGGCAT
Cpp-novel-miR8-3p_supercont3.829:180337-180416	ACACGTCCATTAACTCTGGTAC
Cpp-novel-miR9-1_supercont3.156:169435-169530	AATCGGAATTCTAAAACGGAA
Cpp-novel-miR10_supercont3.329:155673-155749	TGATCTTGTATTTTGATGCTCC
Cpp-novel-miR11-3p_supercont3.1328:35831-35912	CAGTGCATGGCCAACACGGTTT
Cpp-novel-miR12_supercont3.8:2074722-2074801	GGTGTTCACTGCCGGCCTGTATG
Cpp-novel-miR13supercont3.787:29538-29623	GTTAGTTTTGGGCGGGTTTTAGT
Cpp-novel-miR14-3p_supercont3.196:659156-659228	GGCGCGAGCGTGTGTTATTC
Cpp-novel-miR15_supercont3.829:126138-126232	ATTTGTGGTATATGTCGGACGAG
Cpp-novel-miR16-3p_supercont3.112:109706-109789	AAGGAGTGGAACTTGGTCGCGGA
Cpp-novel-miR17-3p_supercont3.57:559449-559540	CGGGATTCCAACTGATATCCAC
Cpp-novel-miR18-3p-1_supercont3.1369:75814-75893	TTGCAGTGGATGGTCGTTTGACG
Cpp-novel-miR19-3p-1_supercont3.1422:16129-16229	CAGGAGTTGATTTGGAGGACACCA
Cpp-novel-miR20-1_supercont3.533:194362-194441	TGATTGTTTACACTCGATCGTTGG

Cpp-novel-miR21-1_supercont3.1761:8353-8427	TTGTCAGTGACGGGTAGTTAGGTT
Cpp-novel-miR21-3_supercont3.207:246904-246978	TTGTCAGTGACGGGTAGTTAGGTT
cqu-miR-8-5p_MIMAT0014408	CATCTTACCGGGCAGCATTAGA
cqu-miR-2941-2-5p_MIMAT0016971	TGAATTTCGCTGGAGTTCTGCCGGA
cqu-miR-980_MIMAT0014428	TAGCTGCCTAGTGAAGGGC
cqu-miR-13_MIMAT0014368	TATCACAGCCATTTTGACGAGT
cqu-miR-2941-1-5p_MIMAT0014434	CGTGGTGTTTAGTCGTAGTGC
cqu-miR-252-3p_MIMAT0014374	CCTGCTGCCCAAGTGCTTATCG
cqu-miR-281-3p_MIMAT0014359	TGTCATGGAATTGCTCTCTTT
cqu-miR-2951-5p_MIMAT0014432	AGAGCTCAGCACGCAGGGGTGGC
cqu-miR-10-5p_MIMAT0014404	ACCCTGTAGATCCGAATTTGTT
cqu-miR-305-5p_MIMAT0014382	ATTGTACTTCATCAGGTGCTCT
cqu-miR-998_MIMAT0014445	TAGCACCATGAGATTCAGC
cqu-bantam-5p_MIMAT0014420	CCGGTTTTCATTTTCGATCTGAC
cqu-miR-315_MIMAT0014365	TTTTGATTGTTGCTCAGAAAGC
cqu-miR-277_MIMAT0014371	TAAATGCACTATCTGGTACGAC
cqu-miR-125-3p_MIMAT0014356	TCACAAGTTTTGATCTCCGGTAT
cqu-miR-1889-3p_MIMAT0014441	CACGTTACAGATTGGGGTTTCC
cqu-miR-71-3p_MIMAT0014401	TCTCACTACCTTGTCTTTCATG
cqu-miR-988-5p_MIMAT0014362	TGTGTGCTTTGTGACAACGAGA
cqu-miR-124_MIMAT0014360	TAAGGCACGCGGTGAATGC
cqu-miR-1175-3p_MIMAT0014391	TGAGATTCTACTTCTCCGACT
cqu-miR-100-3p_MIMAT0014388	CAAGGCCGGATATATGGGATC
cqu-miR-275_MIMAT0014364	TCAGGTACCTGAAGTAGCGC
cqu-miR-210-5p_MIMAT0014396	AGCTGCTGACCACTGCACAAGA
cqu-miR-276-5p_MIMAT0014392	AGCGAGGTATAGAGTTCCT
cqu-miR-9-3p_MIMAT0014381	TAAAGCTTTAGTACCAGAGGTC
cqu-miR-12-5p_MIMAT0014394	AAGAATGAAAATCCTGCCCTGTCGGAT
cqu-miR-2951-3p_MIMAT0014433	ACCCGCGTCTACCCGGTTCCGTGTACTGGAAAT
cqu-miR-279-5p_MIMAT0014417	GATGGGTGTGAATCTAGTGGTTC
cqu-miR-31-3p_MIMAT0014426	AGCTATTCAACTTCTTGTCTAT
cqu-miR-308_MIMAT0014429	AATCACAGGAGTATACTG
cqu-miR-285-5p_MIMAT0014410	ACTGGCTTCCAAAGGTGAGTAGA
cqu-let-7-3p_MIMAT0014367	CTATGCAATCCGCTAGCTTAAC
cqu-bantam-3p_MIMAT0014421	TGAGATCATTTTGAAAGCTGA
cqu-miR-9-5p_MIMAT0014380	TCTTTGGTATTCTAGCTGTAGA
cqu-miR-79_MIMAT0014430	GCTTTGGCGCTTTAGCTGTATGA
cqu-miR-305-3p_MIMAT0014383	CGGCACATGTTGGAGTACAC
cqu-miR-11-5p_MIMAT0014406	CGAGAACTCCGGCTGTGACC

cqu-miR-932-5p_MIMAT0014414	TCAATTCCGTAGTGCATTGCAG
cqu-miR-317-5p_MIMAT0014384	CGGGATACACCCTGTGCTCGCTTTGC
cqu-miR-8-3p_MIMAT0014409	TAATACTGTCAGGTAAAGATGT
cqu-miR-993-3p_MIMAT0014376	GAAGCTCGTTTCTATAGAGG

Table S2. Primers used for qRT-PCR validation of differentially expressed miRNAs.

**Target miRNA**	**Primer Name**	**Original Species used for primer design**	**Qiagen Catalog Number**
miR-8-5p	dme-miR-8-5p	*Drosophila melanogaster*	YP02110716
miR-92a	aga-miR-92a	*Anopheles gambiae*	YP02109225
miR-1891	aae-miR-1891	*Aedes aegypti*	YP02111773
miR-2941-3p-1	aae-miR-2941-3p-1	*Aedes aegypti*	YP02101472
miR-283	cqu-miR-283	*Culex quinquefasciatus*	YP02107736
miR-2952-3p	cqu-miR-2952-3p	*Culex quinquefasciatus*	YP0211919
novel-miR-1	cpp-novel-miR-1	*Culex pipiens*	YCP2151324
Novel-miR-4	cpp-novel-miR-4	*Culex pipiens*	YCP2151345
let-7	aga-let-7	*Anopheles gambiae*	YCP0201184

Table S3. Read count summary for the six samples (three each from *Cx. p.pipiens* and *Cx. p. molestus*) at sequential steps of processing for differential expression analysis.

Name	Filename	Number of raw reads	Number of cleaned reads	Percent surviving read cleaning	Contaminant alignment rate	Size sorted reads	Counts mapped to miRNAs	Percent mapped (Size sorted/Mapped miRNA)
Molestus Rep 1	M1_S4_L001_R1_001.fastq.gz	62925125	51097708	81.20%	0.49%	23769110	19459880	81.87%
Molestus Rep 2	M2_S5_L001_R1_001.fastq.gz	59171062	50303619	85.01%	0.45%	27960485	22117141	79.10%
Molestus Rep 3	M4_S6_L001_R1_001.fastq.gz	80251815	66316405	82.64%	0.39%	36760493	28481455	77.48%
Pipiens Rep 1	P1_S1_L001_R1_001.fastq.gz	48470972	38366161	79.15%	0.32%	15546853	7868115	50.61%
Pipiens Rep 2	P2_S2_L001_R1_001.fastq.gz	89622867	72875986	81.31%	0.47%	31743584	15543979	48.98%
Pipiens Rep 3	P3_S3_L001_R1_001.fastq.gz	67928714	56724825	83.51%	0.32%	24728313	11227141	45.40%

Table S4. All miRNAs that were analyzed in DESeq2. Log2 fold change is written as *Cx. p. pipiens* relative to *Cx. p. molestus* (i.e., positive values are upregulated in *Cx. p. pipiens*). The *p*-value has been adjusted with a Benjamini–Hochberg false discovery rate. miRNAs from Hong et al. (2014) are named additionally with position information (i.e., super contig location) and miRNAs from miRbase are labeled with identifiers from the miRbase database ("MIMAT").

miRNA	log2FoldChange	Adjusted p-value
Cpp-miR-iab-4_supercont3.12:681153-681228	-0.0148735	0.61508364
cqu-miR-12-5p_MIMAT0014394	-0.0150171	0.6043417
cqu-miR-2941-1-5p_MIMAT0014434	-0.0158699	0.49056312
cqu-let-7-3p_MIMAT0014367	-0.062678	0.396748
cqu-miR-1175-3p_MIMAT0014391	-0.0201756	0.37671411
Cpp-miR-375-1_supercont3.4:25124-25212	0.07059022	0.63586403
Cpp-miR-309-3p_supercont3.145:66026-66116	-0.1861714	0.14512211
Cpp-miR-965-3p_supercont3.48:484122-484208	0.07058757	0.70523189
Cpp-miR-33_supercont3.1258:69328-69403	0.00499203	0.94372916
cqu-miR-308_MIMAT0014429	-0.0615975	0.70523189
Cpp-miR-993_supercont3.12:55477-55565	-0.0868118	0.6191713
cqu-miR-79_MIMAT0014430	-0.1382208	0.52724362
Cpp-miR-117_supercont3.86:866106-866187	-0.123497	0.07407613
Cpp-novel-miR13supercont3.787:29538-29623	0.20348454	0.396748
Cpp-miR-1174_supercont3.86:865891-865964	-0.0703328	NA
Cpp-novel-miR4_supercont3.153:639486-639575	2.3203493	4.71E-09
cqu-miR-317-5p_MIMAT0014384	0.04081528	0.83104214
cqu-miR-279-5p_MIMAT0014417	0.04191834	0.83104214
cqu-miR-988-5p_MIMAT0014362	0.25152906	0.32749395
Cpp-miR-2952-3p_supercont3.5:753920-753983	-3.2989007	4.71E-09
Cpp-miR-996_supercont3.19:1437000-1437085	0.22072083	0.39485799
cqu-miR-993-3p_MIMAT0014376	-0.0455838	0.82955614
cqu-miR-285-5p_MIMAT0014410	-0.1258742	0.6043417
Cpp-novel-miR12_supercont3.8:2074722-2074801	0.35721759	0.14792726
Cpp-miR-2941-3p-1_supercont3.5:753632-753715	-2.4020312	1.56E-05
cqu-miR-252-3p_MIMAT0014374	0.26291198	0.28357293
Cpp-novel-miR1_supercont3.64:982778-982859	1.28596365	4.31E-11
cqu-miR-980_MIMAT0014428	-0.5204627	0.07407613
cqu-miR-125-3p_MIMAT0014356	0.181707	0.396748
Cpp-miR-79-3p_supercont3.83:80579-80662	0.02587468	0.8712229
cqu-miR-31-3p_MIMAT0014426	0.14494783	0.49848962
cqu-miR-1889-3p_MIMAT0014441	0.34248247	0.10467865

cqu-miR-9-3p_MIMAT0014381	-0.1303317	0.5718105
Cpp-miR-316_supercont3.496:152458-152539	0.18713324	0.40059563
cqu-miR-305-5p_MIMAT0014382	-0.2818398	0.14792726
Cpp-miR-988-3p_supercont3.791:14282-14361	0.05736725	0.79445264
Cpp-miR-989-3p_supercont3.315:321306-321394	-0.1565857	0.04874439
cqu-miR-210-5p_MIMAT0014396	-0.2851568	0.16081872
cqu-miR-305-3p_MIMAT0014383	0.45619191	0.01419726
cqu-miR-100-3p_MIMAT0014388	0.51417183	0.00196792
cqu-miR-276-5p_MIMAT0014392_precursor_cqu-mir-276-1_MI0013589	0.41143395	0.07407613
Cpp-miR-1890_supercont3.64:982778-982862	-0.1092148	0.58937108
Cpp-miR-10-3p_supercont3.12:95990-96072	0.1724042	0.42796907
Cpp-miR-92a_supercont3.722:174902-174982	-0.617176	0.00044481
cqu-miR-281-3p_MIMAT0014359	-0.4604905	0.14248426
Cpp-miR-190-1_supercont3.181:347902-347986	-0.0314533	0.8712229
cqu-bantam-5p_MIMAT0014420	0.05462501	0.77175808
cqu-miR-932-5p_MIMAT0014414	-0.4389772	0.01777675
Cpp-miR-12-3p_supercont3.153:639666-639741	-0.2859872	0.07646622
cqu-miR-11-5p_MIMAT0014406	0.27364422	0.22390308
Cpp-miR-31_supercont3.559:256532-256609	0.1434023	0.49848962
Cpp-miR-133-3p_supercont3.1189:55672-55785	-0.2983559	0.25846607
Cpp-miR-281-1_supercont3.1661:13247-13325	-0.0929924	0.70523189
Cpp-miR-210-3p_supercont3.549:157647-157724	-0.2337368	0.25846607
Cpp-novel-miR8-3p_supercont3.829:180337-180416	0.1815228	0.4118657
Cpp-miR-932-3p_supercont3.261:301401-301481	0.11492599	0.6043417
Cpp-miR-283_supercont3.57:559440-559549	-0.7015704	0.00196792
Cpp-miR-315_supercont3.438:61916-61999	-0.0852691	0.70523189
cqu-miR-9-5p_MIMAT0014380	-0.0507126	0.79445264
cqu-miR-276-5p_MIMAT0014392_precursor_cqu-mir-276-2_MI0013589	-0.0668978	0.73124415
Cpp-miR-1000_supercont3.153:102798-102891	-0.1095273	0.60342782
cqu-miR-124_MIMAT0014360	-0.2554452	0.23137843
Cpp-miR-278-3p_supercont3.16:1026152-1026243	-0.0398239	0.83104214
Cpp-miR-137-3p_supercont3.1714:27556-27636	-0.0178669	0.8712229
cqu-miR-998_MIMAT0014445	-0.0716825	0.66261803
Cpp-miR-981_supercont3.431:151310-151408	0.02574181	0.8712229
cqu-miR-71-3p_MIMAT0014401	-0.2790116	0.16081872
Cpp-miR-71_supercont3.366:117493-117583	0.23856402	0.17018156
cqu-miR-10-5p_MIMAT0014404	0.04852141	0.77175808
cqu-miR-8-5p_MIMAT0014408	-0.6891858	0.00013146

cqu-miR-2951-5p_MIMAT0014432	-0.064826	0.77175808
Cpp-miR-2951-1_supercont3.1464:4408-4477	-0.0520302	0.79445264
Cpp-miR-87_supercont3.431:379743-379819	0.20001051	0.396748
Cpp-miR-1891_supercont3.829:180333-180418	0.7857169	0.00044481
Cpp-miR-125_supercont3.4:280965-281043	-0.215164	0.24996278
Cpp-miR-306_supercont3.83:80431-80502	0.02444773	0.87122995
cqu-miR-2951-3p_MIMAT0014433	-0.2087002	0.294092
Cpp-miR-970-3p_supercont3.495:35917-36003	0.0548937	0.77175808
cqu-miR-13_MIMAT0014368	-0.1232124	0.52724362
cqu-miR-275_MIMAT0014364	-0.1253092	0.46808512
Cpp-miR-279-3p_supercont3.19:1441114-1441197	-0.216909	0.22390308
Cpp-miR-2-3p-1_supercont3.366:116579-116662	-0.1721902	0.39485799
Cpp-miR-317-3p-1_supercont3.36:1133161-1133241	-0.0529633	0.73124415
Cpp-miR-957-3p_supercont3.787:29537-29622	0.23571302	0.18614261
Cpp-miR-11-3p_supercont3.153:639659-639743	0.0477329	0.79445264
Cpp-miR-252-1_supercont3.1787:6793-6867	0.10104541	0.62219398
Cpp-miR-1889_supercont3.57:562539-562677	-0.1062147	0.5718105
cqu-miR-277_MIMAT0014371	-0.1172643	0.52724362
Cpp-miR-100_supercont3.4:271404-271484	0.1383665	0.50749308
Cpp-miR-263_supercont3.219:351799-351880	0.15090835	0.50291049
cqu-bantam-3p_MIMAT0014421	0.0944189	0.66903324
cqu-miR-8-3p_MIMAT0014409	0.04567819	0.8095424
Cpp-miR-14-3p_supercont3.676:52241-52327	0.13546855	0.5718105
Cpp-bantam_supercont3.65:199694-199763	0.48154673	0.00044481
Cpp-miR-285-3p_supercont3.98:262280-262362	0.04740575	0.79445264
Cpp-miR-184-3p_supercont3.567:240302-240382	0.29343489	0.14792726
Cpp-miR-276-3p-1_supercont3.136:340857-340942	0.02330862	0.8712229
Cpp-miR-999-3p_supercont3.14:96865-96948	-0.1201188	0.60424963
Cpp-miR-1-3p_supercont3.78:246202-246281	-0.0824759	0.70523189
Cpp-miR-7_supercont3.1:3357343-3357422	0.30763015	0.06410465

Table S5: Validation that miRNAs are significantly, differentially expressed in independent samples of biting Pipiens (positive fold change) and non-biting Molestus (negative fold change) when measured with either RNAseq or qRT-PCR. Cpp-miR-29252-3p was detected with qRT-PCR in 4/5 biological replicates of non-biting Molestus, but not in any biological replicates of biting Pipiens.

miRNA	RNA seq Results		qRT-PCR Results	
	Log2 Fold Change	Adjusted p-value	Log2 Fold Change	p-value
Cpp-miR-2952-3p	-3.3091	3.79E-09	*	*
Cpp-miR-2941-3p	-2.4171	1.08E-05	-1.924	0.006892
Cpp-miR-283	-0.7057	0.00173	-0.6721	0.0201
Cpp-miR-8-5p	-0.7008	7.40E-05	-0.7158	0.00464
Cpp-miR-92a	-0.6245	0.0004123	-0.6081	0.0114
Cpp-miR-1891	0.7867	0.0004123	0.3405	0.1814
Cpp-novel-miR-1	1.278	5.14E-11	1.467	4.47E-05
Cpp-novel-miR-4	2.311	4.56E-09	1.782	0.0241

Figure S1. Principle components plot of gene expression profiles in *Cx. p. molestus* (non-biting) *Cx. p. pipiens* (biting) samples. m (red) dots = biological replicate samples of *Cx. p. molestus* (non-biting), p (aqua) dots = biological replicate samples of *Cx. p. pipiens* (biting).

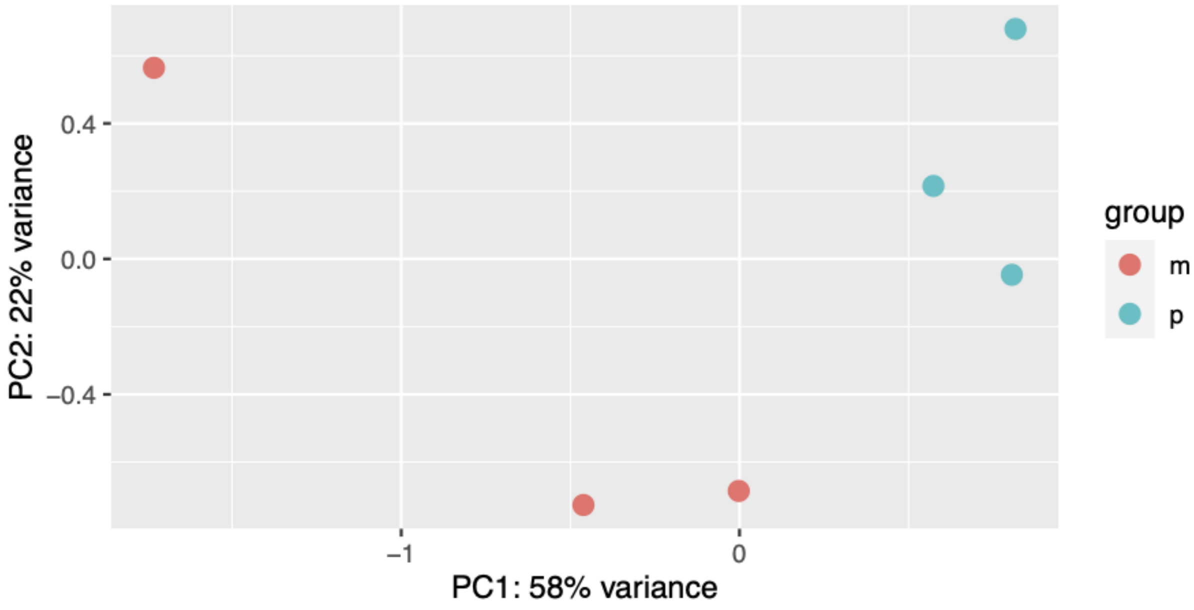

Supplement: Supplementary file 1 [file insects-14-00700-s001.zip › Marzec et al_Insects_SM.pdf]
